# Retention‐in‐care in the PMTCT cascade: definitions matter! Analyses from the INSPIRE projects in Malawi, Nigeria and Zimbabwe

**DOI:** 10.1002/jia2.25609

**Published:** 2020-10-08

**Authors:** Helene Font, Nigel Rollins, Shaffiq Essajee, Renaud Becquet, Geoff Foster, Alexio‐Zambezio Mangwiro, Victor Mwapasa, Bolanle Oyeledun, Sam Phiri, Nadia A Sam‐Agudu, Nita B Bellare, Joanna Orne‐Gliemann, for the INSPIRE group

**Affiliations:** ^1^ ISPED, Inserm Bordeaux Population Health Research Center UMR 1219 University of Bordeaux Bordeaux France; ^2^ Department of Maternal, Newborn, Child and Adolescent Health World Health Organization Geneva Switzerland; ^3^ Data and Analytics Section Unicef New York USA; ^4^ Family AIDS Caring Trust World Health Organization Mutare Zimbabwe; ^5^ Clinton Health Access Initiative Harare Zimbabwe; ^6^ Department of Public Health College of Medicine University of Malawi Blantyre Malawi; ^7^ Centre for Integrated Health Programs Abuja Nigeria; ^8^ Lighthouse Trust Lilongwe Malawi; ^9^ Department of Medicine University of North Carolina School of Medicine Chapel Hill NC USA; ^10^ Department of Public Health School of Public Health and Family Medicine College of Medicine University of Malawi Lilongwe Malawi; ^11^ International Research Center of Excellence Institute of Human Virology Nigeria Abuja Nigeria; ^12^ Division of Epidemiology and Prevention Institute of Human Virology University of Maryland School of Medicine Baltimore MD USA; ^13^ Strategic Information Department UNAIDS Geneva Switzerland

**Keywords:** HIV care continuum, retention, Africa, women, PMTCT, outcome assessment

## Abstract

**Introduction:**

Definitions of retention‐in‐care in Prevention of Mother‐to‐Child Transmission of HIV (PMTCT) vary substantially between studies and programmes. Some definitions are based on visits missed/made, others on a minimum total number of visits, or attendance at a final clinic visit at a specific time. An agreed definition could contribute to developing evidence‐based interventions for improving retention‐in‐care. In this paper, we estimated retention‐in‐care rates according to different definitions, and we quantified and visualized the degree of agreement between definitions.

**Methods:**

We calculated retention in care rates using nine definitions in the six INSPIRE PMTCT intervention studies, conducted in three sub‐Saharan African countries between 2013 and 2017. With data from one of the studies (E4E), we estimated the agreement between definitions using Gwet’s agreement coefficient (AC1) and concordance. We calculated positive predictive values (PPV) and negative predictive values (NPV) for all definitions considering successively each definition as the reference standard. Finally, we used a Multiple Correspondence Analysis (MCA) to examine clustering of the way different definitions handle retention‐in‐care.

**Results:**

Retention‐in‐care rates among 5107 women ranged from 30% to 76% in the complete dataset with Gwet’s AC1 being 0.56 [0.53; 0.59] indicating a moderate agreement between all definitions together. Two pairs of definitions with high inner concordance and agreement had either very high PPV or very high NPV, and appeared distinct from the other five definitions on the MCA figures. These pairs of definitions were also the ones resulting in the lowest and highest estimates of retention‐in‐care. The simplest definition, that only required a final clinic visit to classify women as retained in care, and classified 55% of women as retained in care, had a PPV ranging from 0.7 to 1 and a NPV ranging from 0.69 to 0.98 when excluding the two pairs afore‐mentioned; it resulted in a moderate to substantial agreement and a 70% to 90% concordance with all other definitions.

**Conclusions:**

Our study highlights the variability of definitions in estimating retention‐in‐care. Some definitions are very stringent which may be required in some instances. A simple indicator such as attendance at a single time point may be sufficient for programme planning and evaluation.

## 1 INTRODUCTION

Lifelong antiretroviral treatment (ART) for mothers living with HIV is critical for the Prevention of Mother‐to‐Child Transmission of HIV (PMTCT) [1]. ART benefits women’s own health and survival and reduces HIV transmission risks for the child [2, 3]. To achieve these, health services must reliably deliver care at every step of the PMTCT cascade [4, 5] and women must continue to attend facilities, that is be “retained in care” [6, 7, 8, 9].

Measures of “retention‐in‐care” are sometimes used as a proxy for adherence to ART interventions and ultimately viral suppression [10]. However, there is currently no gold standard definition or metric of PMTCT retention‐in‐care [11, 12, 13, 14]. Concepts such as loss to follow‐up [15, 16], engagement in care [17] or linkage to care [18] have been used to describe women’s attendance and retention in care; these are clearly related but have different inferences and implications. A recent study comparing five definitions of point retention‐in‐care at 12‐month post ART initiation, using the same dataset, found that rates varied from 1.2% to 98% [19]. Similarly, when using different data sources to estimate various definitions, another study showed that rates of retention‐in‐care varied from 41% to 72% [20]. Both studies underline the importance of carefully choosing the retention‐in‐care definition as the basis for analyses, and of using different definitions on the same dataset [21].

Because of such variability, comparing rates of retention‐in‐care between research or programmes is difficult and impedes the development of evidence‐based guidelines and evaluating the effectiveness of interventions by settings. Yet, little is reported about how definitions of retention‐in‐care are related and how to choose between them.

We examined facility‐attendance data and definitions applied in six intervention studies (known collectively as INSPIRE) that aimed to improve retention‐in‐care among mothers living with HIV in three sub‐Saharan African countries. INSPIRE was an implementation research initiative launched in 2012 by the World Health Organization (WHO) with the goal of testing and integrating effective PMTCT interventions within existing health services [22]. Specifically, our objectives were to estimate rates of retention‐in‐care according to different definitions, and second to quantify the degree of agreement between retention‐in‐care definitions.

## 2 METHODS

### 2.1 Study setting

INSPIRE included five cluster‐randomized controlled trials and one prospective cohort study conducted between 2013 and 2017 in Malawi (PURE and PRIME), Nigeria (MoMent and LJM) and Zimbabwe (E4E and EPAZ). Each study implemented different intervention packages, focused on the improvement of the local health system and/or the implementation of peer‐support programmes. All six studies investigated retention‐in‐care as their main outcome, however, they applied different definitions of retention‐in‐care. Study designs and population characteristics are described elsewhere [23, 24, 25, 26, 27, 28].

### 2.2 Study samples

For the first objective, aiming at estimating rates of retention‐in‐care according to different definitions, we used data of all pregnant and post‐partum women enrolled into the six studies, from both control and intervention arms. Women who withdrew from the studies were excluded.

For the second objective, exploring agreement and differences between retention‐in‐care definitions, we used data from one study only, in order to reduce variance due to differences in data collection methods, inclusion/exclusion criteria or national PMTCT protocols. We used the E4E study data, as it had a large sample size, high completeness of data regarding dates of scheduled appointments.

### 2.3 Data sources

All studies used routine facility data that were abstracted by research staff from pre‐natal and post‐natal clinic registers or patient cards. These data were captured into study‐specific databases and later extracted and merged for the purpose of these analyses.

Clinic visit dates that were not directly related to prescribing or reviewing of ART were excluded for consistency. With the exception of one study, the date of clinic visit by a pregnant woman or mother was accompanied by the date of the next scheduled appointment. Since follow‐up duration varied between studies, we restricted the analysis to the data consistently available, that is one year after study enrolment.

### 2.4 Retention‐in‐care definitions

Nine definitions of retention‐in‐care were used in the analyses (Table 1). Six definitions were from the INSPIRE studies: four were based on missed visits [29, 30, 31, 32]; one on the number of clinic visits [33]; and one on attendance at a final clinic visit [34]. We also used three additional definitions of retention‐in‐care identified in the literature and formulated around other concepts: visit constancy [35]; gaps in care [36]; and the number of isolated clinic visits [13], which is a South African measure (Health Resources and Services Administration HIV/AIDS Bureau) [37].

**Table 1:** Retention‐in‐care definitions

#	Definitions	Reference	Country	N	Definition based on
1	Being in care at 335 days post‐delivery or later and no missed visits (>14 days of the scheduled appointment)	[30]	Malawi	1350	Missed visits
2	Final visit (six‐months postpartum ± 30 days) and no missed visits (>30 days of the scheduled appointment)	[31]	Nigeria	532	
3	No missed visits (≥60 days of the scheduled appointment)	[32]	Malawi	1269	
4	Being in care at 335 days post‐delivery or later and <25% of missed visits (>14 days of the scheduled appointment) + no gap in care >90 days	[29]	Zimbabwe	1150	
5	Attending at month 12 post‐delivery [±1 month]	[34]	Zimbabwe	350	Final visit
6	Attending ≥4 times	[33]	Nigeria	497	Number of visits
7	Having ≥1 HIV clinic visit every three months	[35]	USA	782	Visit constancy
8	Time interval between completed clinic visits <3 months	[36]	Ethiopia	346	Gaps in care
9	Having completed ≥2 visits separated by ≥3 months within a 12 months period	[37]	USA	—	Number of isolated visits

### 2.5 Statistical analyses

We reclassified each woman, when feasible, according to each retention‐in‐care definition.

For the first objective, we calculated retention‐in‐care rates according to each definition for each study sample and overall. We did not stratify results by control and intervention arms. We applied the study‐specific methodology for handling missing “scheduled appointment date” (e.g. case deletion or imputation). The names of the six INSPIRE studies were replaced by labels (A to F) as the aim of these analyses is not to compare rates between specific studies but to examine the importance of chosen definitions on the variability of retention‐in‐care rates.

For the second objective, we used different statistical methods on the E4E dataset, using case deletion for missing data. The Gwet’s first‐order agreement coefficient (AC1) [38, 39] score was computed to assess the degree of agreement among pairs of definitions in the classification of women as retained or not retained. AC1 scores were interpreted as “Excellent agreement” for scores over 0.80, “Substantial agreement” for 0.61 to 0.80, “Moderate agreement” for 0.41 to 0.60, “Fair agreement” for 0.21 to 0.40 and “Slight agreement” under 0.21 [40]. We also estimated the percentage of concordant classifications between pairs of definitions (i.e. the proportion of women that two definitions similarly classified as retained or not retained). Furthermore, we calculated the Positive predictive values (PPV) and Negative predictive values (NPV) of all retention‐in‐care definitions. As none of the definition is considered a gold standard, PPVs/NPVs were calculated, successively using each definition as the reference standard for the eight others. PPV therefore corresponds to the proportion of women retained per the reference standard among those retained by the definition assessed. NPV corresponds to the proportion of women who were considered as not retained per the reference standard among those not retained by the definition assessed. Lastly, to further understand concordance and differences between retention‐in‐care definitions, we conducted a Multiple Correspondence Analysis (MCA) considering, for each woman in the E4E study, the retention‐in‐care status according to the nine definitions. MCA is a descriptive and exploratory method used to visualize how observations, most often patients, are clustered according to multiple qualitative characteristics, and according to un‐measured observed “dimensions” [41]. We used it here to visualize the clustering of retention‐in‐care definitions according to individual status. As for individual MCAs, we interpreted factors that may explain this clustering.

### 2.6 Ethics

All studies were approved by their respective national Ethics Committee and the World Health Organization Ethics Review Committee. EPAZ, LJM, MoMent, PRIME and PURE studies obtained written consent before enrolment. E4E study used de‐identified data abstracted from registers and did not required individual consent. The Clinical Trials Registration numbers were NCT02070900 (E4E), NCT02216734 (EPAZ), NCT02214875 (LJM), NCT01936753 (MoMent) and NCT02005835 (PURE). The PRIME study registered with Pan African Clinical Trial Registry PACTR201312000678196. These analyses only used de‐identified data.

## 3 RESULTS

### 3.1 Study population characteristics

After excluding 41 women who withdrew, a total of 5107 women living with HIV from all six INSPIRE studies contributed to the analyses. Median age of enrolled women was 28 years (IQR = 23 to 32) and 12.9% (N = 604) were primipara. Among the 4 360 women who enrolled while pregnant and with a known gestational age at enrolment (85% of the full sample), the majority booked during the second semester of pregnancy (N = 3 131; 72%), 526 (12%) booked late during the third trimester and 703 (16%) booked during the first semester of pregnancy. Regarding ART initiation, 4242 women (83%) were not on ART at the time of first booking (a high percentage, in part, because it was an inclusion criterion in some INSPIRE studies). Among women with a known delivery outcome (N = 4 059), 3 889 (96%) had a live birth.

For the second analysis exploring the agreement and concordance between retention‐in‐care definitions, we only used the E4E study population of 1150 women (N = 1 073 after case deletion). Their median age was 26 years (IQR = [22 to 31]) and 22% (N = 233) were primipara. Of these, 68% (N = 719) booked for antenatal care during the second semester of pregnancy, 15% (N = 156) booked later during the third semester and 17% (N = 180) booked early during the first semester of pregnancy. All were ART naïve at enrolment. Live birth rate was 95% with 814 live births among 861 known delivery outcomes.

### 3.2 Retention‐in‐care rates according to different definitions

Retention‐in‐care rates for the INSPIRE study populations, estimated according to the nine definitions, are presented in Table 2. Global retention‐in‐care rates ranged from 30% (with definition #1 “No missed visit defined by two weeks after an appointment”) to 76% (with definition #9 “number of isolated clinic visits”). The variability in rates was even greater when different definitions were applied to individual study datasets, for example rates for study population B ranged between 12% and 79% according to the definition used.

**Table 2:** Retention‐in‐care rates for each of the six INSPIRE study populations and combined, according to the nine definitions

#	Definitions	INSPIRE All n = 5107	Study A sample	Study B sample	Study C sample	Study D sample	Study E sample*	Study F sample*
1	No missed visit – 14 days	29.9	32.6	11.6	32.7	31.6	*–*	*–*
2	No missed visit – 30 days	38.1	38.0	23.8	43.3	38.7	*–*	*–*
3	No missed visit – 60 days	63.0	62.8	52.3	68.9	61.4	*–*	*–*
4	<25% missed visit	54.8	55.6	41.6	60.0	53.7	*–*	*–*
5	Final visit	55.0	56.4	60.7	59.9	51.7	67.2	31.8
6	Number of visits	70.3	66.3	75.7	76.9	74.4	80.2	42.3
7	Visit constancy	53.0	49.6	56.6	63.1	56.4	62.9	17.9
8	Gap in care	43.4	39.4	48.9	52.4	43.9	53.7	17.5
9	Number of isolated visits	75.9	73.0	79.3	80.8	77.0	84.8	58.8

* One study did not collect scheduled appointment dates and another had a high proportion of women with at least one missing scheduled appointment dates. As this variable was necessary to compute definitions based on missed visit, rates for these definitions are missing for two studies.

The variability of the estimated retention‐in‐care rates across different studies also differed according to the definition used. Focusing on study samples A to D (i.e. excluding the samples with missing data), we observed that retention‐in‐care rates varied little with the definition based on the number of isolated clinic visits (#9), from 73% to 81%. However, when using the “No missed visit defined by two weeks after an appointment” definition (#1), the difference of retention‐in‐care rates was higher, from 12% to 33%.

### 3.3 Agreement and concordance between retention‐in‐care definitions

**Figure 1:**
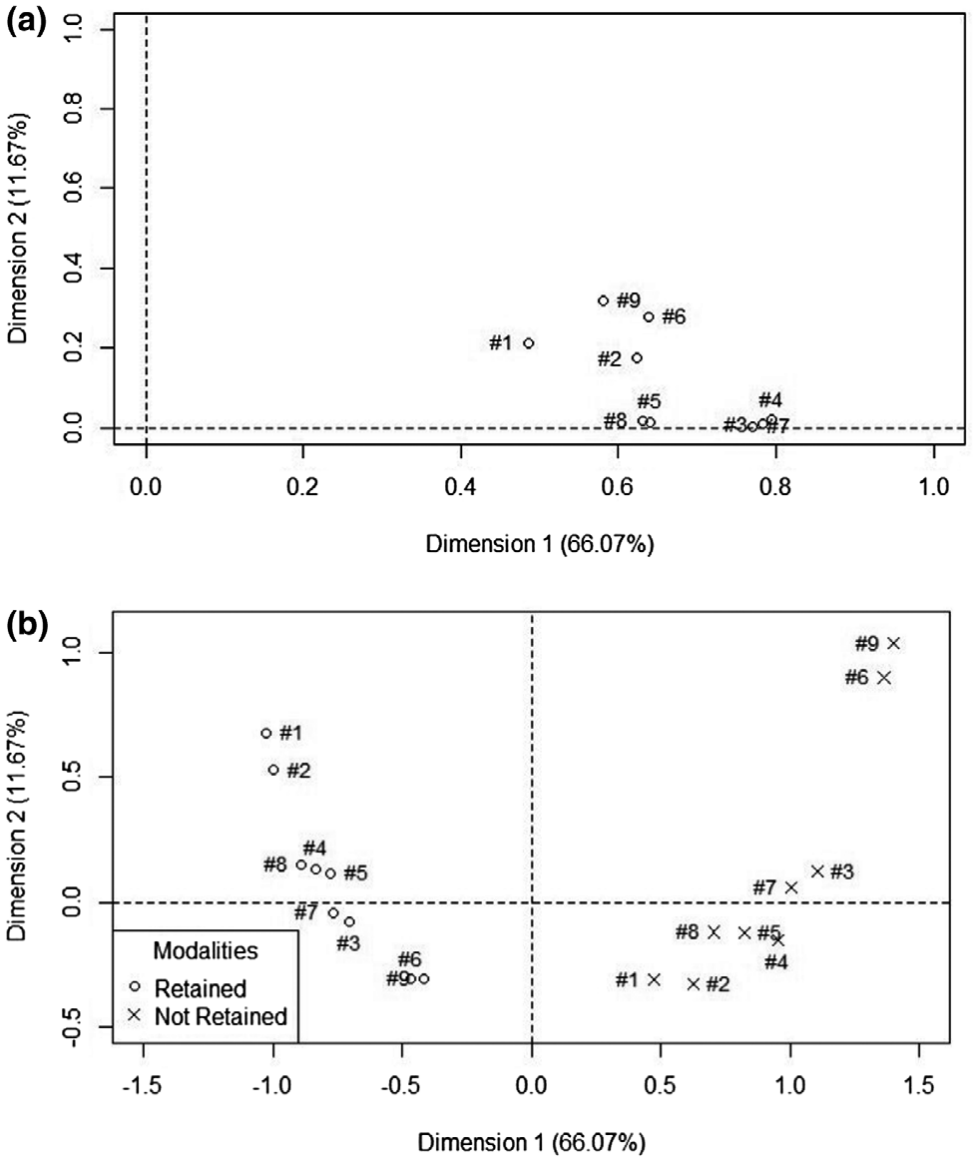
Retention‐in‐care definitions and modalities: Representation of the two first dimensions of the MCA (66% and 12% of inertia respectively). (a) Representation of the definitions. (b) Representation of the response modalities.

The agreement (Gwet’s AC1 score – top right of the table) and concordance (% ‐ bottom left of the table) between definitions of retention‐in‐care are shown in Figure 1. The overall Gwet’s AC1 was 0.56 [0.53; 0.59] indicating a moderate agreement between the nine retention‐in‐care definitions. Pairwise analyses showed a majority of moderate to substantial agreements and concordance levels of 70% to 90%. For example, definitions #5 and #2 had a substantial agreement (AC1 = 0.7) and agreed in categorizing women as “retained” in 85.4% of cases.

The two definitions resulting in the highest rates (#6 and #9) and the two resulting in the lowest rates of retention‐in‐care (#1 and #2) each demonstrated high levels of agreement and concordance (AC1 = 0.9 and 0.7, concordance = 96.7% and 84.8%, respectively). However, agreement and concordance between these two pairs (#6 and #9 vs. #1 and #2) were, at best, only fair (AC1 from 0.1 to 0.3, concordance from 53.8% to 63.5%).

### 3.4 Positive and negative predictive values

The PPV/NPV calculated for the nine definitions are shown in Table 3, with definition #1 to #9 successively considered in each column as the reference standard for PPV/NPV of the eight other definitions considered as diagnostic tests. The highest PPVs (0.83 to 1) were found for definitions #1 and #2 (those resulting in the lowest rates of retention‐in‐care), using any of the other seven as a reference standard; however, definitions #1 and #2 had NPVs ranging from 0.33 to 0.79. In contrast, definitions #6 and #9 (those resulting in the highest retention‐in‐care estimates) had very high NPVs (0.98 to 1) and lower PPVs (ranging from 0.40 to 0.79). Definition #5, the simplest definition, which only required a final clinic visit to classify women as retained‐in‐care, showed PPVs ranging from 0.54 to 1 and NPVs ranging from 0.47 to 0.98. However, when excluding as reference standard the four definitions resulting in the lowest and highest rates of retention‐in‐care (#1, #2, #6 and #9), PPVs for definition #5 ranged from 0.70 to 0.90 and its NPVs ranged from 0.69 to 0.83.

**Table 3:** Positive (PPV) and Negative Predictive Values (NPV) for each retention‐in‐care definition examined with each definition alternatively considered as the diagnostic test and as the reference standard

Definitions		Considered as the reference standard								
		#1	#2	#3	#4	#5	#6	#7	#8	#9
Considered as the test										
#1	PPV		0.87	1	1	0.88	0.99	0.96	0.83	0.99
	NPV		0.84	0.57	0.68	0.66	0.37	0.61	0.74	0.33
#2	PPV	0.71		0.99	0.98	0.98	0.99	0.93	0.82	0.99
	NPV	0.93		0.63	0.75	0.78	0.41	0.66	0.79	0.37
#3	PPV	0.51	0.62		0.84	0.76	0.99	0.88	0.73	1
	NPV	1	0.99		0.95	0.86	0.64	0.93	1	0.58
#4	PPV	0.59	0.71	0.96		0.85	0.99	0.93	0.76	0.99
	NPV	1	0.99	0.79		0.87	0.54	0.85	0.92	0.48
#5	PPV	0.54	0.73	0.9	0.88		0.99	0.87	0.7	1
	NPV	0.93	0.98	0.69	0.83		0.51	0.76	0.83	0.47
#6	PPV	0.42	0.51	0.81	0.71	0.68		0.76	0.59	1
	NPV	0.99	0.99	0.98	0.99	0.98		1	1	0.88
#7	PPV	0.53	0.63	0.95	0.88	0.79	1		0.76	1
	NPV	0.97	0.94	0.84	0.91	0.85	0.59		0.97	0.53
#8	PPV	0.59	0.71	1	0.91	0.81	1	0.97		1
	NPV	0.9	0.88	0.7	0.77	0.72	0.46	0.76		0.41
#9	PPV	0.4	0.5	0.79	0.69	0.66	0.96	0.73	0.57	
	NPV	0.99	0.99	0.99	0.99	0.99	0.99	1	1	

### 3.5 Multiple correspondence analysis

**Figure 2:**
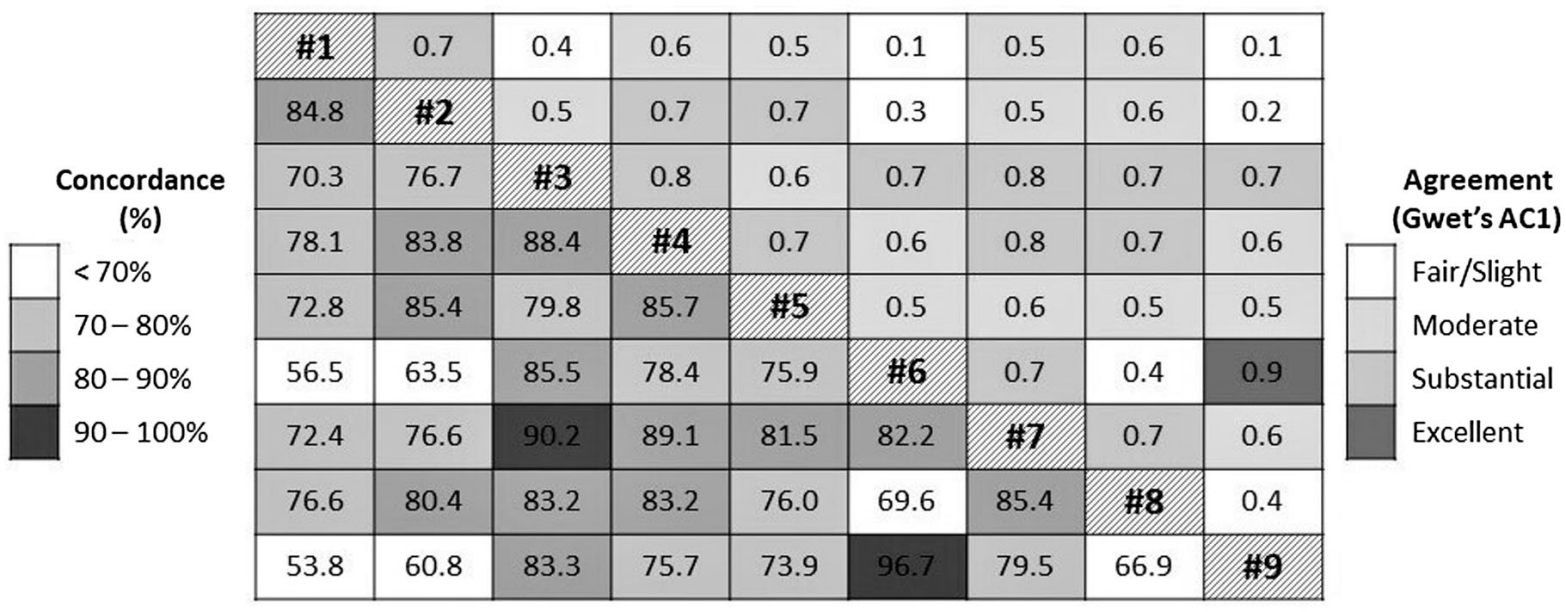
Agreement (right side of the diagonal) and concordance (left side of the diagonal) between retention‐in‐care definitions (using E4E dataset only). Labels of Definitions are in the diagonal. “Excellent agreement” was for scores over 0.80, “Substantial agreement” for 0.61 to 0.80, “Moderate agreement” for 0.41 to 0.60, “Fair agreement” for 0.21 to 0.40 and “Slight agreement” under 0.21 [40].

Findings of the MCA conducted with the nine retention‐in‐care definitions are shown in Figure 2a. The nine definitions, represented as points, are distributed roughly in the same area of the graph. More specifically, they are aligned principally along the x‐axis, which describes the main underlying dimension common to all nine definitions, and that we thus interpreted as a retention‐in‐care dimension. This suggests that, despite differences, all definitions are globally similar in measuring retention‐in‐care. Definitions #1, #2, #6 and #9 however, are grouped slightly apart from the other five along the y‐axis. This suggests that there are some differences in the way these four definitions perform in terms of classifying women as retained or not, as compared to the other definitions. Figure 2b shows in more detail how the retention‐in‐care response relate one to another. The first observation here is that, for all definitions, “retained” is situated on the left on the x‐axis whereas “Not retained” is located to the right, confirming the observation in Figure 2a, that all nine definitions generally concur in classifying women for retention in care. The second observation is that definitions #1 and #2 seems to cluster higher on the y‐axis in the “retained” group, and definition #6 and #9 cluster similarly in the not‐retained group. We interpret this as being consistent with the fact that these two pairs of definitions are similarly more restrictive (#1 and #2) in defining retention in care and more restrictive (#6 and #9) in defining non retention in care.

## 4 DISCUSSION

In this pooled analysis of data from the six INSPIRE implementation research studies, we observed considerable variability in estimated retention‐in‐care rates among pregnant women and mothers living with HIV according to the definitions used. When applied to the same dataset, different definitions yielded rates ranging between 30% and 76%. Some definitions, especially more stringent definitions, appeared to result in greater variability of estimated retention‐in‐care rates according to study settings and populations. Retention‐in‐care definitions based on missed visits, and with stricter criteria (zero missed visits and short intervals between scheduled and actual clinic visits, definitions #1 and #2), not surprisingly, led to the lowest estimates of retention‐in‐care. Conversely, the two definitions based on the number of clinic visits attended (#6 and #9) systematically led to the highest retention‐in‐care rates. This inter‐definition variability suggests that some heterogeneity in retention‐in‐care rates reported in the literature [42, 43] could be attributed to differences in definitions applied, as much as or even more than to differences in intervention efficacy or programme quality. Any retention‐in‐care estimation should thus consider the influence of the definition applied and even explore the effect through sensitivity analyses. Any consensus position, for example determined by UNAIDS or WHO, should consider the purpose of the definition, that is for programme review, national monitoring and comparison or research, and the respective strength and limitation of each definition approach.

The agreement and concordance analyses, as well as the MCA analyses, were consistent in their findings and confirmed the differences between two specific pairs of definitions: those leading to the lowest estimates that were based on the concept of missed visits (#1 and #2) and those leading to the highest estimates that were based on the number of clinic visits (#6 and #9).

Perhaps surprisingly, the definition that was based only on “final clinic visit” (#5) performed very similarly to other definitions. Despite its very simple construct (only using one final clinic visit), definition #5 seemed to capture a large part of the information conveyed by more complicated definitions that emphasized recurrent attendances throughout PMTCT follow‐up. Its PPV and NPV indicate that this simplest definition could reflect the women’s clinic attendance over the preceding 12 months with similar positive and negative predictive value as other definitions. If found to be robust in other analyses, this single data variable would be easy for health systems to prioritize and capture accurately. Others have already advocated for a single clinic visit definition [44] because of its simplicity and that it clearly defines “out‐of‐care patients.” Also, a definition based on a specific clinic visit date may be less susceptible to the quality (sometimes poor) and the availability (also sometimes poor) of routine health data.

Our study had several limitations. First, we explored only a limited number of retention‐in‐care definitions and applied them to a specific research population. Definitions related to linkage to care [18] and loss to follow‐up [16, 45] have been reported from other HIV‐related programmes and may provide valuable insights related to retention‐in‐care. Second, we limited our analyses to a 12‐month period. Exploring the effects of applying retention‐in‐care definitions over longer periods of time, for example 24 or 36 months may reveal different associations; this may include what happens as mothers move out of early postnatal care and into routine child health or ART services. Actual retention‐in‐care may differ according to the stage of care – soon after diagnosis or ART initiation, or in ART care [46, 47]. Finally, the most significant limitation was the lack of other process and clinical data available at the time of analysis. Pill count and HIV viral load data would have allowed further investigation of the relationship between retention‐in‐care and women’s health status. More work is needed to better understand the link between the regular clinic visits during a specific time period and the final clinic visit at the end of this period. Despite these limitations, one of the major strengths of this study is the detailed scrutiny of this key indicator of programme and intervention success through a combination of several methodological approaches, which as triangulation in qualitative analysis, leads to highly valid data.

The variability in retention‐in‐care estimates depending on criteria used highlights the importance of understanding how a definition is constructed and what is its primary purpose. Is it based on missed visits or attendance patterns? Is it intended to facilitate comparisons of interventions as part of implementation research or to track the performance of programmes; or does the data collection process aim to identify early defaulters or at‐risk populations in order to trigger community‐based tracing? Some definitions require more complex data inputs which will have resource requirements; others will provide greater insights on the behaviour of individuals within a population. When referring to retention‐in‐care rates, researchers and programme managers should appreciate the potential for variance according to criteria used. Failure to do so, may bias interpretation and comparison of interventions and strategies aimed at mitigating losses of individuals from treatment programmes. Different types of retention‐in‐care definition and analyses may be needed for research and for programmes.

## 5 CONCLUSIONS

In summary, our findings highlight the variability of estimated PMTCT retention‐in‐care rates depending on which definition is applied to the analyses. In the absence of an agreed gold standard definition for PMTCT retention‐in‐care and methodologies for estimating rates, it is important that studies provide detailed descriptions of their study population, context, data collection and data management processes in order to accurately interpret findings and compare the effectiveness of relevant interventions. In contexts where retention‐in‐care rates may be used to infer ART adherence and to calculate both antenatal and postnatal PMTCT risks, for example as part of modelling work around the estimation of new paediatric HIV infections, the type of definition used by national programmes will be an important point to take into consideration. A simple indicator such as attendance or non‐attendance at a single time point, for example 12 months postpartum may be sufficient for programme planning rather than more detailed and complex indicators that may only be achievable with electronic record systems.

## COMPETING INTERESTS

All authors declare no competing interests.

## AUTHORS’ CONTRIBUTIONS

HF, NR and JOG designed and implemented the analysis. HF and JOG searched the literature and co‐wrote the first draft of the manuscript. HF did the statistical analysis. NR contributed substantive changes to the draft. SE, RB, GF, AM, VM, BO, SP, NS‐A, NB reviewed the draft, contributed to the interpretation and presentation of the findings. All authors have read and approved the final version of the manuscript for submission.

## ABBREVIATIONS

AC1, Gwet’s first‐order agreement coefficient; ART, Antiretroviral treatment; E4E, Evidence‐for‐Elimination study; EPAZ, Elimination of Paediatric AIDS in Zimbabwe study; HRSA HAB, Human Resources and Services Administration HIV/AIDS Bureau; IQR, Inter‐Quartile Range; LJM, Lafiyan Jikin Mata study; MCA, Multiple Correspondence Analysis; MoMent, Mother Mentor study; NPV, Negative predictive values; PMTCT, Prevention of Mother‐to‐Child Transmission; PPV, Positive predictive values; PRIME, Promoting Retention among Infants and Mothers Effectively study; PURE, Prevention of mother‐to‐child transmission Uptake and REtention study.
